# Intractable Pyopneumothorax due to Lemierre's Syndrome Treated With an Endobronchial Watanabe Spigot (EWS)

**DOI:** 10.1002/rcr2.70302

**Published:** 2025-07-30

**Authors:** Mio Sugino, Masao Hashimoto, Sho Morishima, Ayumi Tonsho, Hiroto Hatano, Yutaro Ueki, Mika Iwasaki, Akane Ishida, Go Naka, Shinyu Izumi, Tatsuo Maeyashiki, Satoshi Nagasaka, Masayuki Hojo

**Affiliations:** ^1^ Department of Respiratory Medicine National Center for Global Health and Medicine Tokyo Japan; ^2^ Department of Thoracic Surgery National Center for Global Health and Medicine Tokyo Japan

**Keywords:** bronchopleural fistula, endobronchial watanabe spigot (EWS), intractable pneumothorax, Lemierre's syndrome, pyothorax

## Abstract

Lemierre's syndrome is a rare condition characterised by internal jugular vein thrombophlebitis and bacteremia caused by anaerobic organisms following an oropharyngeal infection. We report the case of a 29‐year‐old woman who presented with fever, sore throat, dyspnea, and chest pain. Chest computed tomography (CT) revealed lung suppuration, pneumothorax, and thrombosis of the left internal jugular vein, confirming the diagnosis of Lemierre's syndrome. During treatment, she developed pyothorax with bronchopleural fistulas and intractable pneumothorax. Although antibiotics and thoracic drainage controlled the infection, persistent air leakage and incomplete lung expansion remained. Placement of an endobronchial watanabe spigot (EWS) successfully resolved the air leak, allowing the patient to avoid surgery and be discharged. This case highlights the effectiveness of EWS in managing intractable pyopneumothorax secondary to Lemierre's syndrome.

## Graphical abstract

This manuscript reports a rare case of Lemierre's syndrome complicated by pyothorax with bronchopleural fistulas and intractable pneumothorax in a 29‐year‐old woman. Despite infection control with antibiotics and thoracic drainage, persistent air leakage required intervention with endobronchial watanabe spigots (EWS), which successfully resolved the air leak and avoided surgery. This is the first reported case of Lemierre's syndrome treated with EWS, highlighting its effectiveness as a minimally invasive option for managing refractory pulmonary complications.

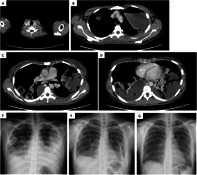

## 1 Introduction

Lemierre's syndrome is a rare condition characterised by internal jugular vein thrombophlebitis and bacteremia, primarily caused by anaerobic organisms following oropharyngeal infection [1]. The lungs are the most common site of metastatic infection, and complications such as lung suppuration, pyothorax, pneumothorax, and occasionally bronchopleural fistulas have been reported [1, 2]. Even when the infection is adequately controlled, pleural thickening from pyothorax can prevent lung re‐expansion, and bronchopleural fistulas may persist [3].

In 1991, Watanabe et al. reported the usefulness of a solid silicone bronchial filler, later developed into the endobronchial watanabe spigot (EWS) in 2000 [4]. EWS is now used to treat intractable pneumothorax, pyothorax with bronchopleural fistula, prolonged postoperative air leaks, and bronchial fistulas to other organs.

Here, we report a case of Lemierre's syndrome complicated by pyothorax with bronchopleural fistulas and intractable pneumothorax. Although infection was controlled with antibiotics and thoracic drainage, persistent air leakage and incomplete lung expansion necessitated further intervention. Placement of EWS successfully resolved the air leak and avoided the need for surgery. To our knowledge, this is the first reported case of Lemierre's syndrome‐associated intractable pyopneumothorax treated with EWS.

## 2 Case Report

A 29‐year‐old woman was referred to our hospital with fever, sore throat, dyspnea, and chest pain. Two weeks before admission, she developed fever and sore throat. One week later, the sore throat subsided, but dyspnea began. Four days prior to admission, she experienced bilateral pleuritic chest pain and worsening dyspnea, prompting a hospital visit. She had no significant medical history or known allergies. She smoked 15–20 cigarettes daily for 9 years and was not taking any medications.

On physical examination, she was alert, with a blood pressure of 88/64 mmHg, heart rate of 141 beats per minute (regular), respiratory rate of 40 breaths per minute, temperature of 41.2°C, and oxygen saturation of 94% on 5 L/min via face mask. Oral hygiene was good, but bilateral tonsillar erythema was noted. Chest auscultation revealed diminished breath sounds and coarse crackles bilaterally.

**FIGURE 1:**
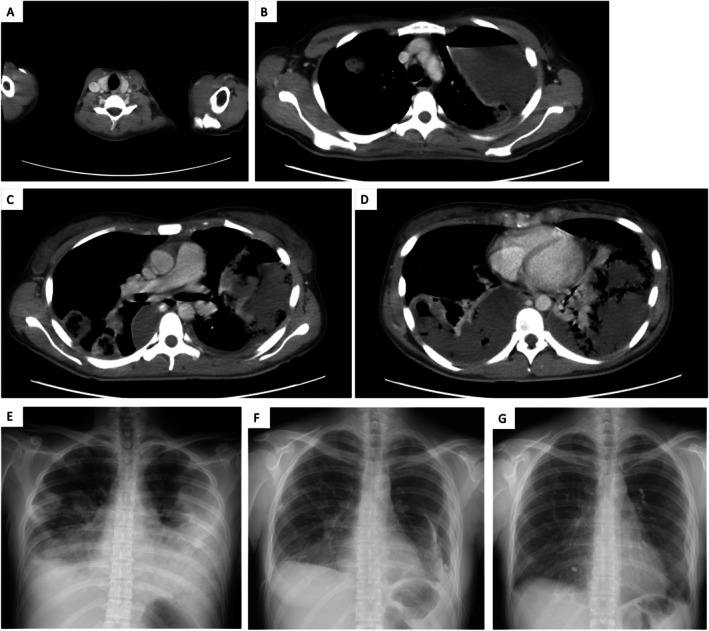
Radiological findings of Lemierre's syndrome and disease progression. (A) Contrast‐enhanced CT on admission showing a thrombus in the left internal jugular vein. (B–D) Axial CT images on admission showing bilateral pleural effusions, with the left effusion encapsulated and containing an air‐fluid level. Numerous cavitary nodules and masses are also visible in both lungs. (E) Chest X‐ray on admission showing bilateral pleural effusions, multiple masses and nodules in both lungs. (F) Chest X‐ray on hospital day 22, prior to EWS placement. Compared with the one on admission, lung suppuration and pyothorax had improved, but new left‐sided pneumothorax was evident. (G) Chest X‐ray on day 106 showing resolution of pneumothorax after EWS treatment.

Laboratory tests (Table 1) on admission revealed a WBC count of 24,000/μL (95% Neutrophils), CRP of 30.34 mg/dL, and D‐dimer of 6.4 μg/mL, indicating severe inflammation. Serum albumin was markedly low at 1.1 g/dL due to inflammatory consumption. Chest X‐ray showed bilateral pleural effusion and multiple pulmonary masses/nodules (Figure 1E). Contrast‐enhanced chest CT revealed a thrombus in the left internal jugular vein (Figure 1A), bilateral pleural effusions with air‐fluid level in the encapsulated left effusion, and numerous cavitary nodules and masses (Figure 1B–D). Thoracentesis yielded pale yellow, purulent, foul‐smelling pleural fluid. Analysis confirmed an exudate with neutrophil predominance and low glucose (Table 1). On hospital day 3, *Fusobacterium necrophorum* was isolated from the pleural fluid. Based on these findings, she was diagnosed with Lemierre's syndrome.

**TABLE 1:** Laboratory findings on admission.

*Haematology*		
RBC	44 × 10^4^	/μL
Haemoglobin	13.1	g/dL
Haematocrit	37.2	%
WBC	24,790	/μL
Neutrophils	95	%
Lymphocytes	2	%
Platelet	11.6 × 10^4^	/μL
*Biochemistry*		
Total protein	5.5	g/dL
Albumin	1.1	g/dL
Total bilirubin	2.4	mg/dL
AST	26	U/L
ALT	21	U/L
γ‐GTP	89	U/L
LDH	208	U/L
ALP	235	U/L
BUN	28.6	mg/dL
Creatinine	0.88	mg/dL
Na	134	mEq/L
Cl	97	mEq/L
K	4.4	mEq/L
*Coagulation*		
PT‐INR	1.18	
APTT	29	s
Fib	746	mg/dL
D‐dimer	6.4	μg/mL
*Serology*		
CRP	0.21	mg/dL
*Pleural effusion*		
Pale yellow		
Purulent		
Putrid		
Total protein	4.2	g/dL
Glucose	1	mg/dL
LDH	26,006	U/L
Total nucleated cell	118 × 10^3^	/μL
RBC	100	/μL
WBC	117 × 10^3^	/μL
Polymorphonuclear leukocyte	81.3	%
Mononuclear leukocyte	18.7	%
*Microbiology*		
Blood culture (2 set)	—	
Sputum culture	Normal flora	
Pleural effusion culture	*Fusobacterium necrophorum*	
Urine culture	—	

Abbreviations: γ‐GTP, γ‐glutamyl transpeptidase; Na, sodium; Cl, chlorine; K, potassium; ALP, alkaline phosphatase; ALT, alanine aminotransferase; APTT, activated partial thromboplastin time; AST, aspartate aminotransferase; BUN, blood urea nitrogen; CRP, C‐reactive protein; Fib, fibrinogen; LDH, lactate dehydrogenase; PT‐NR, prothrombin time international normalised ratio; RBC, red blood cells; WBC, white blood cells.

We initiated empirical therapy with meropenem (MEPM) and performed bilateral thoracic drainage. On Day 8, antibiotics were de‐escalated to ampicillin/sulbactam (ABPC/SBT) based on susceptibility results of *Fusobacterium necrophorum*. Inflammatory markers improved, and serum albumin levels gradually recovered.

**FIGURE 2:**
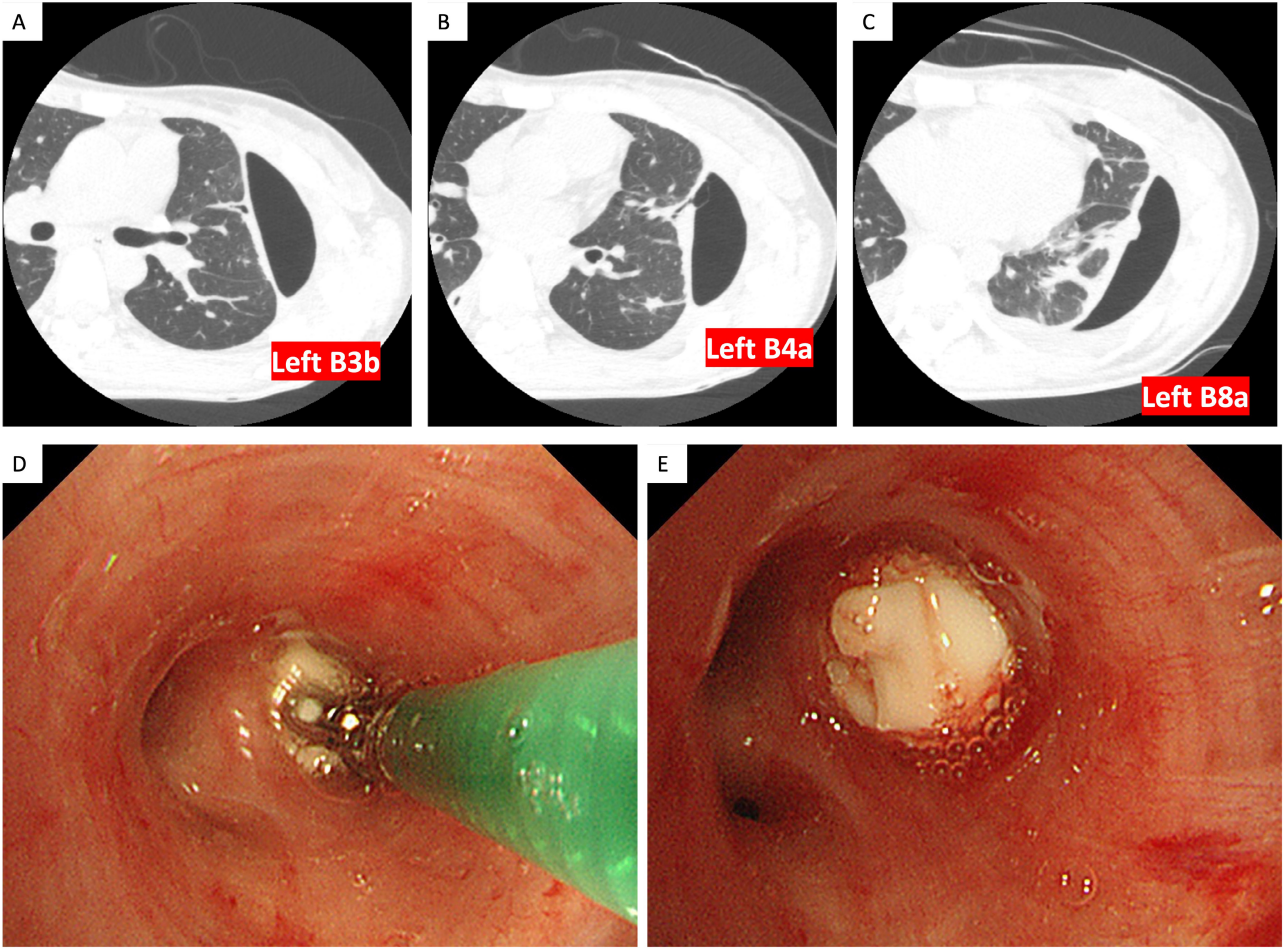
Identification of responsible bronchi and bronchoscopic EWS placement. (A–C) High‐resolution CT (HRCT) on hospital day 36 clearly identified the bronchi responsible for the pneumothorax: left B3b, B4a, and B8a. (D, E) Bronchoscopic findings during EWS placement. EWS were placed in the left B3b, B4a, B8a, and right B10c bronchi.

On Day 21, the patient developed a left‐sided pneumothorax (Figure 1F). Despite continued thoracic drainage, the lung failed to re‐expand, and air leakage persisted. CT identified multiple bronchopleural fistulas as the cause. High‐resolution CT (HRCT) pinpointed the responsible bronchi as left B3b, B4a, and B8a (Figure 2A–C). On Day 44, we inserted endobronchial watanabe spigots (EWS) into these bronchi (Figure 2D,E). A potential bronchopleural fistula was also identified in right B10c, and EWS was prophylactically placed there to avoid compromising right‐lung ventilation if surgery became necessary. Following EWS placement, the air leak resolved and the pneumothorax improved (Figure 1G). We removed the thoracic drain tubes and switched the antibiotics to oral amoxicillin‐clavulanate (AMPC/CVA). She was discharged on hospital day 78 and continued AMPC/CVA for 2 months, during which CT confirmed resolution of lung suppuration and pyothorax. All spigots were removed via bronchoscopy 3 months after placement.

## 3 Discussion

We report a rare case of Lemierre's syndrome complicated by intractable pyopneumothorax, successfully treated with EWS.

Lemierre's syndrome typically involves septic thrombophlebitis of the internal jugular vein after an oropharyngeal infection, with septic emboli often affecting the lungs and joints [1]. The most common causative pathogen is *Fusobacterium necrophorum* , an anaerobic gram‐negative bacillus. Pulmonary manifestations include bilateral infiltrates, pleural effusion, cavitary lesions, and occasionally pneumatoceles, pneumothorax, or acute respiratory distress syndrome (ARDS) [1, 2]. In our case, intractable pneumothorax due to bronchopleural fistulas developed after thoracic drainage, a rare complication [2].

EWS is a minimally invasive option for managing intractable pneumothorax, pyothorax with bronchopleural fistulas, prolonged postoperative air leaks, and bronchial fistulas [4]. While antibiotics and thoracic drainage are first‐line treatments for pyothorax, the presence of bronchopleural fistulas often impairs healing, leading to persistent air leakage and incomplete lung re‐expansion due to pleural thickening [3]. Surgical intervention is typically considered in such refractory cases. However, EWS is a less invasive alternative. In addition to sealing the air leak, EWS prevents the spread of intrapleural infection into the bronchi, facilitates effective thoracic drainage, and may allow improved ventilation in preparation for potential future surgery [5]. Moreover, EWS can be beneficial for older or frail patients who are poor surgical candidates. In the present case, surgical management was under consideration, but EWS placement successfully resolved the air leak, thus avoiding surgery. There have been several reports of using EWS for pyothorax with bronchopleural fistulas caused by various pathogens, achieving air leak resolution and infection control, thus avoiding surgery [6, 7]. Unlike previously reported cases, our case was characterised by Lemierre's syndrome as the underlying cause, which resulted in multiple pleural cavities and multiple bronchopleural fistulas. It was technically demanding to place EWS in several responsible bronchi.

EWS completely stops air leakage in approximately 40% of cases and partially reduces it in another 40% [4]. Identifying the responsible bronchi is crucial for procedural success. HRCT, balloon occlusion testing, pleurography (thoracography), and thoracic dye injection are useful techniques to localise the fistula [8]. In this case, HRCT clearly identified the bronchi, enabling successful targeted EWS placement. Adequate sedation is vital for successful EWS placement, as patient movement and cough can interfere with the procedure. An initial attempt on hospital day 37 under local anaesthesia failed due to excessive motion, but a second attempt under inhalational anaesthesia in an operating room on hospital day 44 was successful and contributed to the favourable outcome.

Although fibrin glue is one of the known materials for bronchial occlusion, EWS has advantages in that it can completely occlude the responsible bronchus and is easily removable if necessary. In addition, a one‐way endobronchial valve is another potential option, but we chose EWS in this case because it is not widely used in Japan and is considered less suitable in the presence of infection.

To our knowledge, this is the first reported case of Lemierre's syndrome complicated by intractable pyopneumothorax successfully managed using EWS. This case illustrates the potential utility of EWS as a therapeutic option in selected patients with such complications of Lemierre's syndrome, emphasising the importance of accurately identifying the responsible bronchi.

## Consent

The authors declare that written informed consent was obtained for the publication of this manuscript and accompanying images and attest that the form used to obtain consent from the patient complies with the Journal requirements as outlined in the author guidelines.

## Conflicts of Interest

The authors declare no conflicts of interest.

## Data Availability

The data that support the findings of this study are available on request from the corresponding author. The data are not publicly available due to privacy or ethical restrictions.
